# Activation of orexin-A (hypocretin-1) receptors in the Raphe Pallidus at different ambient temperatures in the rat: effects on thermoregulation, cardiovascular control, sleep, and feeding behavior

**DOI:** 10.3389/fnins.2024.1458437

**Published:** 2024-10-04

**Authors:** Timna Hitrec, Flavia Del Vecchio, Luca Alberti, Marco Luppi, Davide Martelli, Alessandra Occhinegro, Emiliana Piscitiello, Ludovico Taddei, Domenico Tupone, Roberto Amici, Matteo Cerri

**Affiliations:** ^1^Department of Biomedical and Neuromotor Sciences, University of Bologna, Bologna, Italy; ^2^Department of Neurological Surgery, Oregon Health and Science University, Portland, OR, United States; ^3^Italian Institute of Technology (IIT), Genova, Italy; ^4^National Institute of Nuclear Physics of Bologna, Bologna, Italy

**Keywords:** orexin (hypocretin), rat, raphe pallidus, autonomic nervous system, behavior

## Abstract

The Raphe Pallidus (RPa) is a brainstem nucleus containing sympathetic premotor neurons that control thermogenesis and modulate cardiovascular function. It receives inputs from various hypothalamic areas, including the Lateral Hypothalamus (LH), a heterogeneous region intricately involved in several autonomic and behavioral functions. A key subpopulation of neurons in the LH expresses orexin/hypocretin, a neuropeptide which is crucially involved in the regulation of the wake–sleep states and feeding behavior. The RPa receives orexinergic projections from the LH and orexinergic signalling in the RPa has been shown to enhance thermogenesis in the anaesthetized rat, but only in the presence of an already existing thermogenic drive, without significantly affecting cardiovascular function. The present work was aimed at exploring the effects on thermoregulation and autonomic function and the possible role in the modulation of the wake–sleep states and feeding behavior of orexin injection in the RPa in the free-behaving rat. In order to assess the influence of an already present thermogenic drive on orexinergic signalling in the RPa, animals were studied at three different ambient temperatures (Ta, 10°C, 24°C, and 32°C). We found that orexin injection into the RPa variably affected the wake–sleep states, autonomic functions, motor activity, and feeding behavior, at the different Tas. In particular, in the first post-injection hour, we observed an increase in wakefulness, which was large at Ta 24°C and Ta 10°C and rather mild at Ta 32°C. Deep brain temperature was increased by orexin injection at Ta 10°C, but not at either Ta 24°C or Ta 32°C. Moreover, an increase in mean arterial blood pressure occurred at Ta 24°C, which was probably masked by the high baseline levels at Ta 10°C and was completely absent at Ta 32°C. Finally, an enhancement in feeding behavior was observed at Ta 24°C and 10°C only. In accordance with what observed in anaesthetized rats, orexinergic signalling in the RPa seems to be ineffective in the absence of any thermogenic drive. Moreover, the effects observed on the wake–sleep states and feeding behavior introduce the RPa as a novel player in the central neural network promoting wakefulness and feeding.

## Introduction

The Raphe Pallidus (RPa) is a nucleus located in the lower brainstem, intricately involved in various autonomic functions (Cao and Morrison, 2003; Morrison and Nakamura, 2011). Notably, RPa contains sympathetic premotor neurons that promote thermogenesis and heat conservation through the activation of brown adipose tissue (BAT) and the regulation of cutaneous vasomotion, and somatic premotor neurons that control shivering (Morrison and Nakamura, 2011; Nakamura and Morrison, 2011). Furthermore, the RPa actively contributes to the control of cardiovascular function, by modulating heart rate and cutaneous vasomotion (Cao and Morrison, 2003; Morrison and Nakamura, 2019; Zaretsky et al., 2003).

The role of the RPa in thermogenesis is well established (Morrison et al., 2014; Morrison and Nakamura, 2019; Tupone et al., 2011). In anesthetized animal models, the disinhibition of RPa has been shown to promote non-shivering (Morrison et al., 1999) and shivering (Nakamura and Morrison, 2011) thermogenesis and cutaneous vasoconstriction (Blessing and Nalivaiko, 2001). These findings were substantially confirmed in free-behaving animals, where the disinhibition of RPa neurons, induced by the local administration of the GABA_A_ antagonist bicuculline, leads to cutaneous vasoconstriction and to an increase in blood pressure (Cerri et al., 2010). Conversely, the inhibition of the RPa with the GABA_A_ agonist muscimol in rats maintained at a low ambient temperature (Ta), induces a profound hypothermia accompanied by a relevant reduction in heart rate and behavioral phenotype similar to natural torpor (Cerri et al., 2013).

In rodents, the RPa has been shown to receive inputs from various hypothalamic areas, including the preoptic area (POA), the dorsomedial hypothalamus (DMH), the perifornical area (PeF), and the lateral hypothalamus (LH) (da Conceição et al., 2020; Hitrec et al., 2019; Takahashi et al., 2013; Tupone et al., 2011; Yoshida et al., 2009), which are known to be involved in several autonomic and behavioral functions, such as thermoregulation (Morrison and Nakamura, 2011), wake–sleep cycle regulation (Jones, 2005), as well as the integration of these functions (Cerri et al., 2017).

The role of the LH in thermoregulation has been established: activation of BAT has been shown following the local injection of bicuculline in the LH in anesthetized rats (Cerri and Morrison, 2005), while a clear hypothermia has been observed in free-behaving rats exposed to a low Ta following the local injection in the LH of muscimol (Cerri et al., 2014). Such a reduction in body temperature was accompanied by a promotion of non-rapid eye movement (NREM) sleep with enhanced Delta power and by either a depression or a suppression of wake and rapid eye movement (REM) sleep, respectively, and did not apparently have an impact on cardiovascular function.

A subpopulation of PeF/LH neurons has been shown to specifically produce the neuropeptide orexin/hypocretin. Orexin is expressed in two isoforms A and B, with the first expressing higher affinity for both orexin receptors, Orx_1_R and Orx_2_R (Sakurai et al., 1998). Orexin, which was initially characterized for its role in stimulating appetite and regulating food consumption (De Lecea et al., 1998; Sakurai et al., 1998), plays a crucial role in wake–sleep regulation, since orexinergic activation promotes wakefulness and suppresses both NREM and REM sleep, while orexinergic inhibition decreases wakefulness and increases NREM sleep occurrence (Sasaki et al., 2011). Furthermore, disruption of orexin signalling in the brain causes narcolepsy with cataplexy in humans (Peyron et al., 2000; Pizza et al., 2022), and narcoleptic signs in animal models (Chemelli et al., 1999; Lin et al., 1999). However, there is supporting evidence that orexin plays a critical role in the modulation of several other physiological functions (Sagi et al., 2021) including that of thermogenesis (Madden et al., 2012; Tupone et al., 2014, 2011).

The presence of LH-orexinergic projections to the RPa (Berthoud et al., 2005; Tupone et al., 2011), and the role played by non-orexinergic-LH neurons in the modulation of thermogenesis sparked the interest to explore the role of orexin in the control of thermogenesis for potential clinical applications (Tupone et al., 2014). In anesthetized rats orexinergic RPa-projecting neurons modulate thermogenesis through excitatory signals (Tupone et al., 2011), leading to an increase in BAT activity, that is induced at a low Ta, when a thermogenic drive is already present, but not at thermoneutral or high Tas. This effect is not accompanied by significant cardiovascular changes. Further studies in the free-behaving rat partially confirm these results: when animals were exposed to Ta 22–25°C, microinjections of orexin-A into the RPa did not affect cutaneous vasomotion or BAT thermogenesis but caused profound alterations in heart rate and blood pressure (Luong and Carrive, 2012). Furthermore, rats with ablated orexin neurons exhibited diminished cold defence responses (Mohammed et al., 2016).

These data demonstrate that orexin, through direct efferent projections to the RPa, modulates thermogenesis by acting as an enhancer of an existing thermogenic drive (Tupone et al., 2011). However, studies on the free-behaving animals assessing the role of the orexinergic RPa-projection on wake–sleep modulation, on cardiovascular function, and on how the exposure of animals to different Tas can influence the activation of this thermoregulatory pathway are still missing.

Thus, the aim of the present work was to explore how orexin-A modulates thermogenesis, locomotor activity, feeding behavior, cardiovascular responses and wake/sleep activity through its action on RPa neurons in the free-behaving rat exposed to different Tas.

## Methods

### Animals

Nine male CD Sprague–Dawley rats (250- 300 g), were used (*Charles River, Inc*.; Lecco, Italy). Upon arrival, they were housed under standard laboratory conditions (12 h:12 h light–dark cycle, lights on at 09:00, 100 lux at cage level; Ta, 24°C; food and water *ad libitum*). The study protocol, designed to minimize the number of animals used, was approved by the Ethical Committee for Animal Research at the University of Bologna and the Italian Ministry of Health (authorization No. 186/2013-B). The procedures adhere to the guidelines outlined in the European Union (2010/63/UE) and the Italian Ministry of Health (January 27, 1992, No. 116) directives. Oversight was provided by the Central Veterinary Service of the University of Bologna and the National Health Authority, ensuring compliance with ethical standards and the responsible use of animals.

### Surgery

Previously established procedures were used (Hitrec et al., 2021). Briefly, rats were pre-anesthetized with Diazepam (Valium Roche, 5 mg/kg intramuscular) and anesthetized with Ketavet (Ketamine-HCl, Parke-Davis, 100 mg/kg intraperitoneal). During the surgical procedure, the following devices were implanted: (i) a femoral catheter (PA-C40, DataSciences International, St. Paul, MN, United States) for telemetric recording of arterial blood pressure (ABP) and determination of heart rate (HR); (ii) a thermistor (B10KA303N, Thermometrics Corporation, Northridge, CA, United States) enclosed in a stainless-steel needle, stereotactically positioned above the left anterior hypothalamus (from bregma: −2 mm LL, −2 mm AP, −5.5 mm DV) for recording of deep brain temperature (Thy); (iii) electrodes for the recording of electroencephalographic (EEG) and nuchal electromyographic (EMG) activity; (iv) a microinjection guide cannula (C315G-SPC Plastics One Inc., Roanoke, VA, United States), stereotactically placed in the RPa (from lambda: −3 mm AP, 0 mm LL -9.5 DV, Paxinos and Watson, 2007). Following surgery, rats were given a subcutaneous injection of saline (20 mL/kg), an intramuscular injection of wide spectrum antibiotic (ampicillin 100 mg/kg and amikacin 10 mg/kg) and a subcutaneous injection of the analgesic Carprofen (5 mg/kg, *Rimadyl, Zoetis*). During the recovery time, rats were monitored daily for signs of pain and distress, and administered with 5 mg/kg of analgesic when deemed necessary. Following recovery from surgery, rats were housed in a thermoregulated and sound-attenuated recording box for habituation, at standard laboratory conditions, for 2 days.

### Experimental design

**Figure 1:**
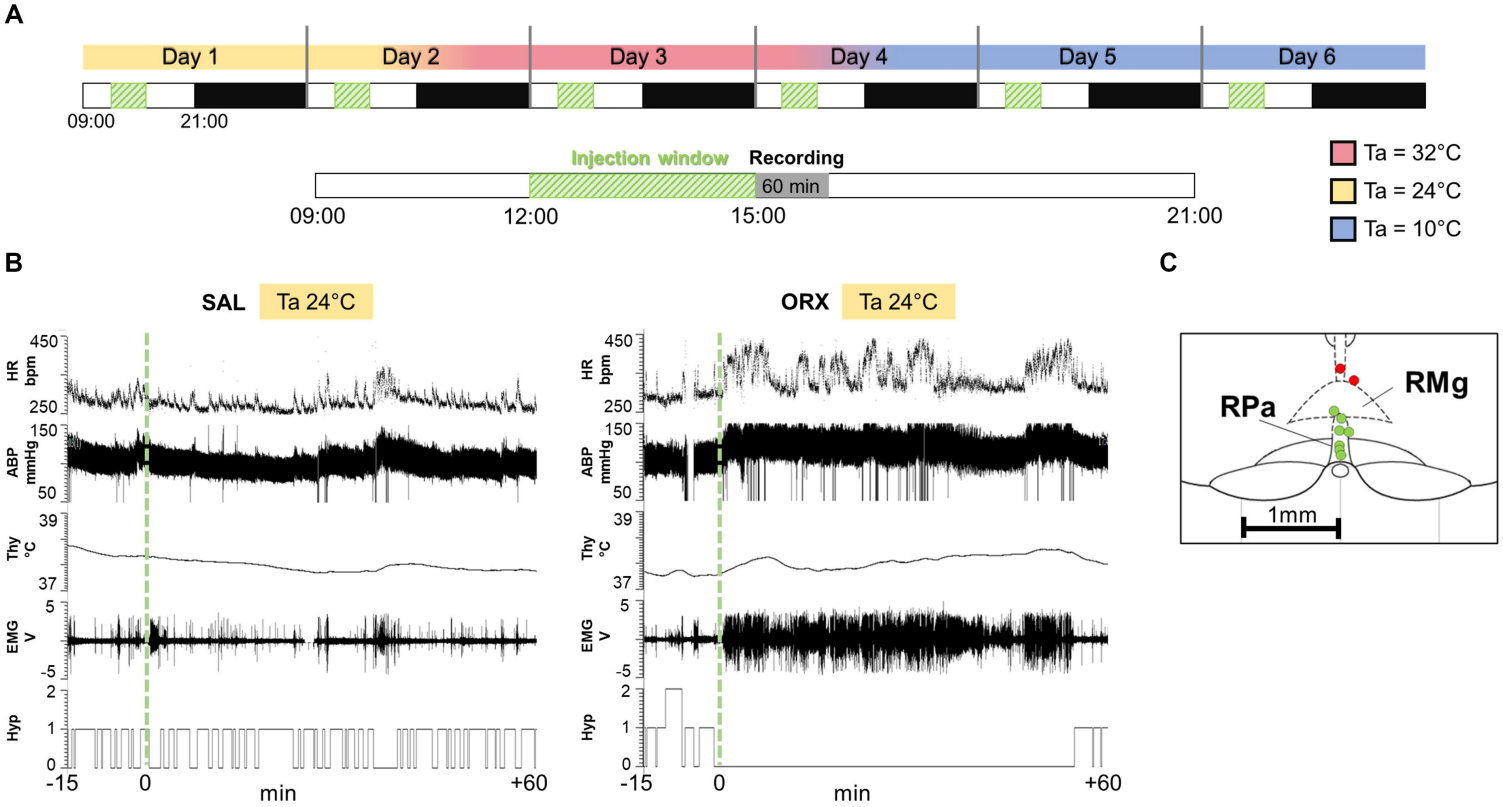
**(A)** Experimental protocol: each animal went through six consecutive days of recording, with sequential exposure to three different ambient temperatures (Ta, 24°C, 32°C, 10°C), which were modified every 2 days (LD cycle 12 h:12H, lights on at 09:00). On each experimental day, the rat received a single injection of either saline (SAL) or the neuropeptide orexin (ORX), administered in a randomized design across the different Tas. The injections were carried out between 12:00 and 15:00, when the rat was particularly quiet, followed by 60 min of variables recording. On the days when a change in ambient temperature was scheduled, this was done 4 h after the injection. **(B)** Example recording of physiological variables 15 min before the injection and 60 min after (injection time = green dashed line). HR = heart rate, ABP = arterial blood pressure, Thy = hypothalamic temperature, Hyp = Hypnogram (0 = wake, 1 = non-REM sleep, 2 = REM sleep), EMG = nuchal electromyographic activity. **(C)** Location of every injection site (n = 9; labelled with a circle, correct injection sites in green, out-of-target in red), marked with Fast Green at the end of each experimental procedure, is schematically plotted on atlas drawings [modified from (Paxinos and Watson, 2007)] to illustrate the distribution and location of RPa injection sites.

For each animal (*n* = 9), the experimental protocol consisted of six consecutive days (Figure 1) of recording with a sequential exposure to three different Ta (24°C, 32°C, 10°C; two consecutive days at each Ta). The order of exposure to the different Tas was maintained constant in order to avoid the potential long-lasting effects of the low Ta on autonomic variables (Chambers et al., 2000). On each experimental day, the rat received a single injection of either saline (SAL) or the neuropeptide orexin-A (ORX) (30 pmol, 150 nL, dissolved in saline, *Tocris Bioscience,* Cat. No. 1455), administered in a randomized design across the different Ta, so that some animals always received ORX on the first day of exposure, while others received it on the second day. The injections were carried out between 12:00 and 15:00, during a time window when the rat was particularly quiet. In fact, since rats display ultradian basic rest–activity cycle (BRAC), where they alternate behaviorally active and inactive states every 1-2 h (Blessing et al., 2013), the injections were made when the rats were in their behaviorally inactive phase. On the days when a change in Ta was scheduled, this was done 4 h after the injection (see Figure 1). Microinjections were performed with previously established procedures (Di Cristoforo et al., 2015). Briefly, a Hamilton 5 μL gastight syringe, placed on an infusion pump (MA 01746, Harvard Apparatus, Holliston, MA, United States) and connected to the internal cannula via one-meter long microdialysis FEP tubing (Microbiotech/se AB, Stockholm, Sweden) was used. The cannula and tubing were filled with either saline or orexin. Thirty minutes after the insertion of the internal cannula, the microinjection was performed using the infusion pump, with an infusion rate of 0.3 μL/min.

During the entire duration of the experiment, a thermal camera (Thermovision A20; FLIR Systems) to record cutaneous temperature as an index of cutaneous vasomotion, and a passive infrared detector (Siemens, PID 10) to measure motor activity based on the movement within the cage, were placed above the cage. Moreover, rats were monitored with a recording camera to assess their behavior. Since it emerged that many animals consumed food soon after the injection, feeding behavior was assessed by analysing the video records and was scored in yes/no dichotomous variables, based on the presence of a feeding episode in the 60 min following the injection (recorded duration of feeding – minimum: 2 min, maximum: 36 min, average: 17 min).

At the end of the experiment, the accuracy of the microcannula placement within the RPa was confirmed through microinjection of Fast Green dye. Subsequently, the animals underwent perfusion with 10% formalin to preserve the tissue. The extracted brains were coronally sectioned to a thickness of 35 μm using a cryostat. As shown in Figure 1, among the nine animals, seven exhibited precise microcannula placement, while two displayed a slightly dorsal injection. Consequently, these two animals were excluded from the study, resulting in a final total of *n* = 7 animals for the analysis.

### Signal processing

Signals were acquired and processed as previously described (Luppi et al., 2017). Briefly EEG, EMG, and Thy were amplified (Grass 7P511L, Astro-Med Inc., West Warwick, RI, United States), filtered (EEG: highpass 0.3 Hz, lowpass 30 Hz; EMG: highpass 100 Hz, lowpass 1 KHz; Tb: highpass 0.5 Hz), and digitalized (Micro MK 1401 II, CED, Cambridge, UK; acquisition rate: EEG: 1 KHz; EMG: 1 KHz; Tb: 100 Hz). Telemetric recording of the ABP signal was amplified and saved on a hard drive (acquisition rate: 500 Hz). For data analysis, ABP was considered as the mean BP value at one-second resolution. HR was calculated based on ABP peak detection as previously described (Cerri et al., 2014). Digital images from the thermal camera were acquired at 1 frame/s and the temperature of the tail (Ttail) was measured in the medial portion of the tail by analysing the thermographic images. The analysis of the EMG signal was made after full-wave rectification of the signal.

Sleep stages were manually scored by an operator with 1-s resolution, utilizing a custom script designed for Spike2 (sleep score). Wakefulness, NREM sleep, and REM sleep were classified according to established criteria based on EEG, EMG, and brain temperature signals (Cerri et al., 2005; Franken et al., 1991). The minimum duration for a wake–sleep episode was set to 4 s for Wake and 8 s for NREM and REM sleep, based on previously established criteria (Cerri et al., 2005).

### Statistical analysis

Statistical analysis was performed using SPSS 21.0, Prism (GraphPad) and Microsoft Excel. At each Ta, a paired t test was used to compare the baseline levels of the wake–sleep states, autonomic parameters, and motor activity in the 30-min period before the injection of either saline or orexin. After assessing that there were no statistically significant differences between baselines levels, we performed a three-way ANOVA for repeated measures to compare the 60 min (six 10-min. bins: 0–10, 10–20, 20–30, 30–40, 40–50, 50–60) following the injection (time 0) of either saline or orexin at the three different Tas (24°C, 32°C and 10°C). Several pre-planned non-orthogonal contrasts were conducted using the modified t-test (t*), with the alpha level adjusted with the “sequential” Bonferroni correction (Holm, 1979).

The factor “time” was considered a within-group factor, with six levels (0–10, 10–20, 20–30, 30–40, 40–50, 50–60 min). Between group factors were: (i) “ambient temperature,” with three levels: 24°C, 32°Cand 10°C, and (ii) “treatment,” with two levels: SAL and ORX.

Feeding behavior was analysed with the Wald Chi-Squared Test, with between groups factors categorized as: (i) “ambient temperature,” with three levels: 24°C, 10°C, 32°C, and (ii) “treatment,” with two levels: SAL and ORX. Differences were considered statistically significant when *p* < 0.05.

## Results

The administration of orexin in the Raphe Pallidus (RPa) induced clear changes at both a behavioral and an autonomic level.

### Effects on wake–sleep states

**Figure 2:**
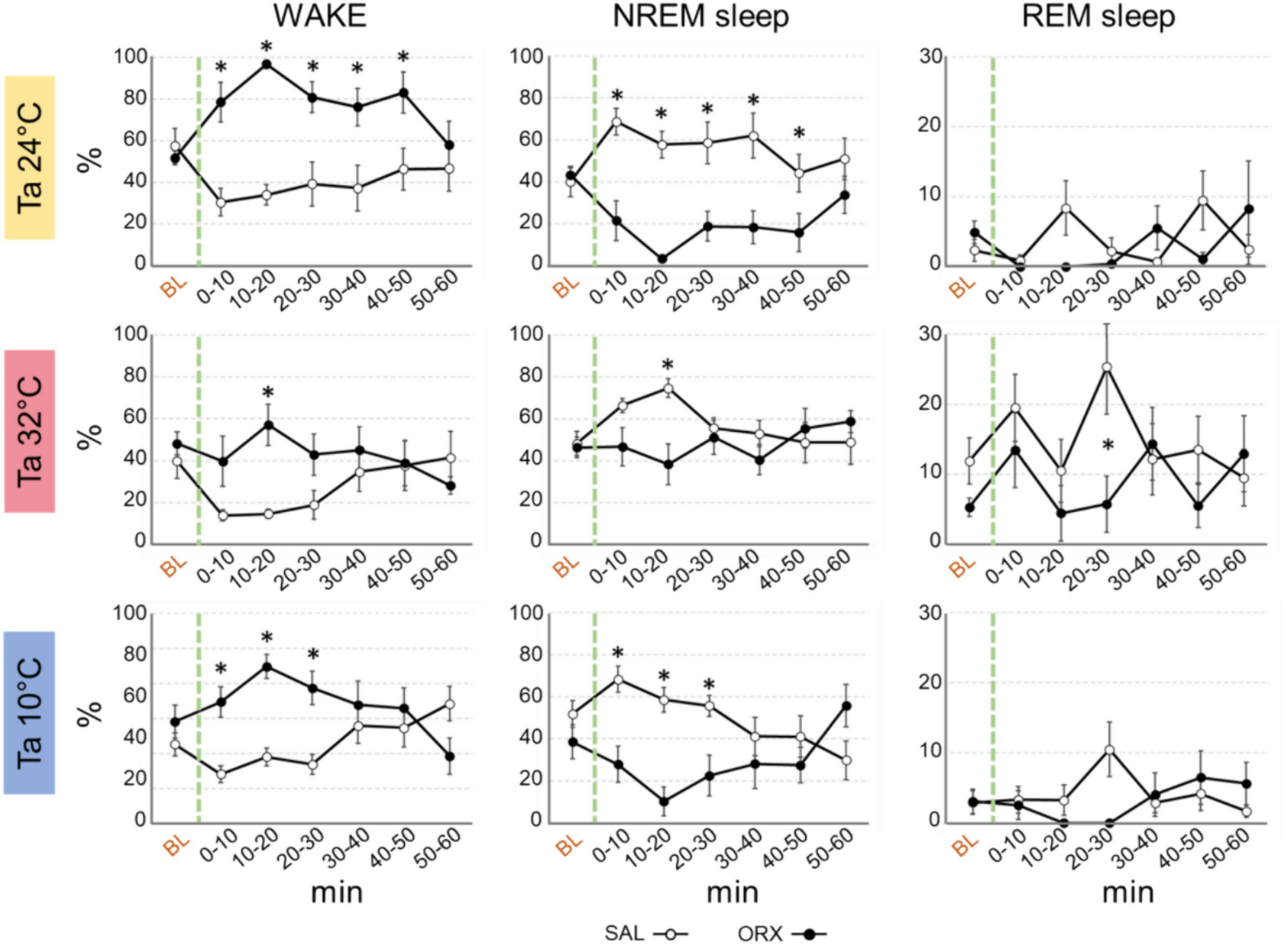
Graphs depicting the mean percentage amount ± S.E.M. of wake, NREM sleep and REM sleep, for the 60 min after the injection (green dashed line) of either saline (SAL, empty dots), or orexin (ORX, filled dots) in the Raphe Pallidus, at three different ambient temperatures (Ta). BL = baseline, indicates the mean percentage value ± S.E.M. of the 30 min prior to the injection. The 60 min following the injection are showed in 10-min time bins. * = *p* < 0.05, green dashed line = injection time.

Regardless of Ta, administration of orexin in RPa caused relevant changes in the wake–sleep states with an increase in the percentage of time spent in wakefulness during the first post injection-hour (Figure 2) when compared to SAL. The clearest effect was observed at Ta 24°C and Ta 10°C, where such an increase was very large and lasted at statistically significant levels for 50 min following the injection at Ta 24°C and between 0 and 30 min after the injection at Ta 10°C. Such an increase was much less evident at Ta 32°C, where peaked and reached statistical significance only in the 10–20 min time window.

Reciprocally, orexin administration caused a significant decrease in NREM sleep, that lasted for the same time duration observed for the increase in wakefulness at either Ta 24°C, Ta 32°C, or Ta 10°C.

The amount of REM sleep appeared to be less affected by orexin administration. There were no significant differences between ORX and SAL at both Ta 24°C and 10°C, whereas at Ta 32°C a significant decrease in REM sleep was observed only between 20 and 30 min after the injection.

### Effects on autonomic variables

**Figure 3:**
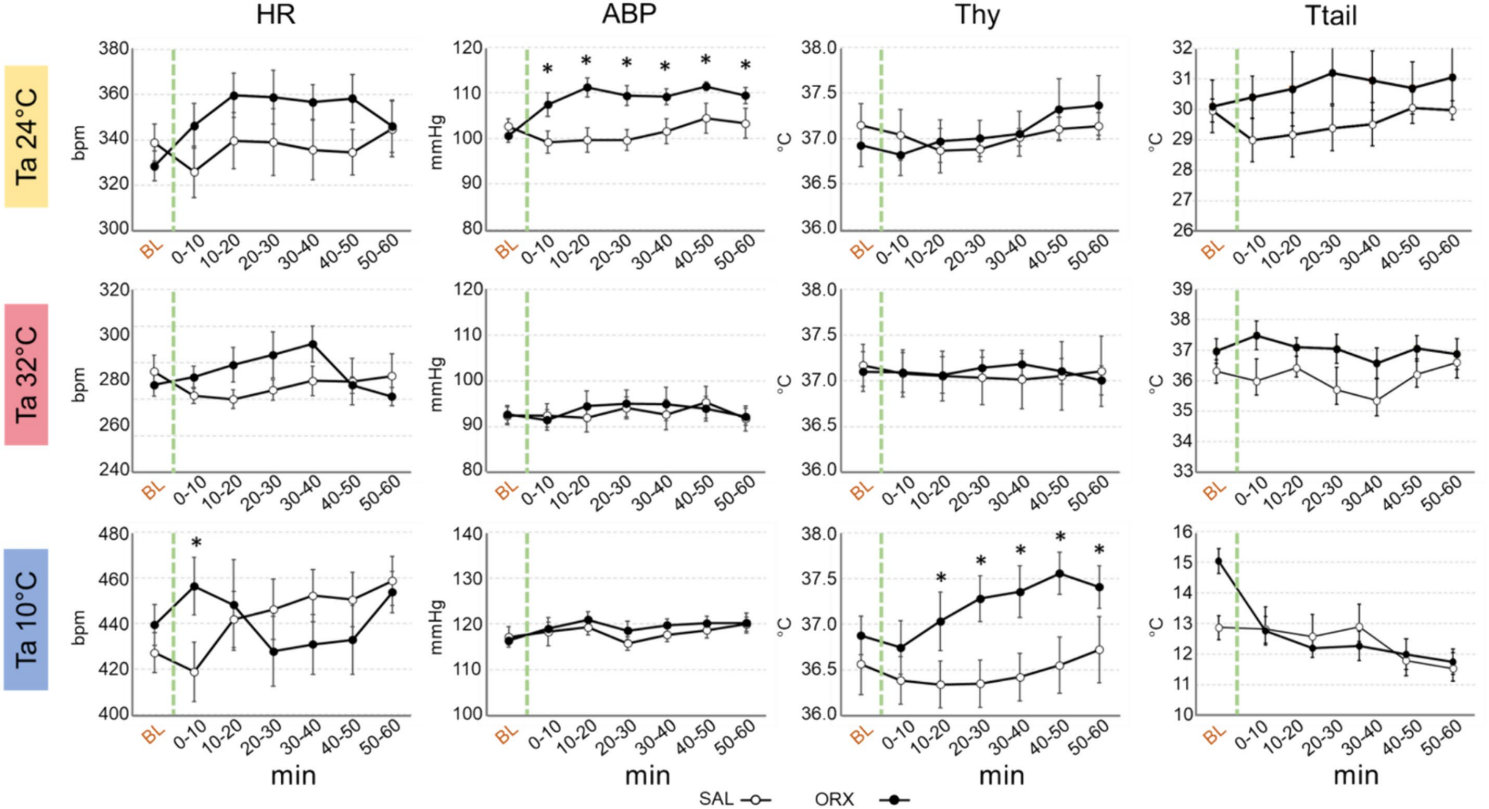
Graphs depicting the mean value ± S.E.M. of heart rate (HR), arterial blood pressure (ABP), hypothalamic temperature (Thy) and tail temperature (Ttail), for the 60 min after the injection (green dashed line) of either saline (SAL, empty dots), or orexin (ORX, filled dots) in the Raphe Pallidus, at three different ambient temperatures (Tas). BL = baseline, indicates the mean value ± S.E.M. of the 30 min prior to the injection. The 60 min following the injection are showed in 10-min time bins. * = *p* < 0.05, green dashed line = injection time.

Compared to the observed changes in wake–sleep states, the autonomic effects of the administration of orexin in the RPa were less prominent and occurred only at Ta 24°C and Ta 10°C (Figure 3). No statistically significant differences between treatments were observed in HR at the different Ta, possibly due to the relatively high variability within groups, except for one time point at Ta 10°C. Orexin administration caused a significant increase in ABP at Ta 24°C, that lasted for 60 min after the injection. No differences were observed at either Ta 10°C or Ta 32°C.

Thy was not significantly affected by orexin administration at both Ta 24°C and Ta 32°C, however a significant increase in Thy was observed at Ta 10°C compared to SAL, that begun 10 min after orexin injection and lasted for the whole recording period.

Ttail did not show any significant change when comparing SAL vs. ORX at any Ta, suggesting that orexin administration in RPa does not affect cutaneous vasomotion in rats.

### Effects on motor activity

**Figure 4:**
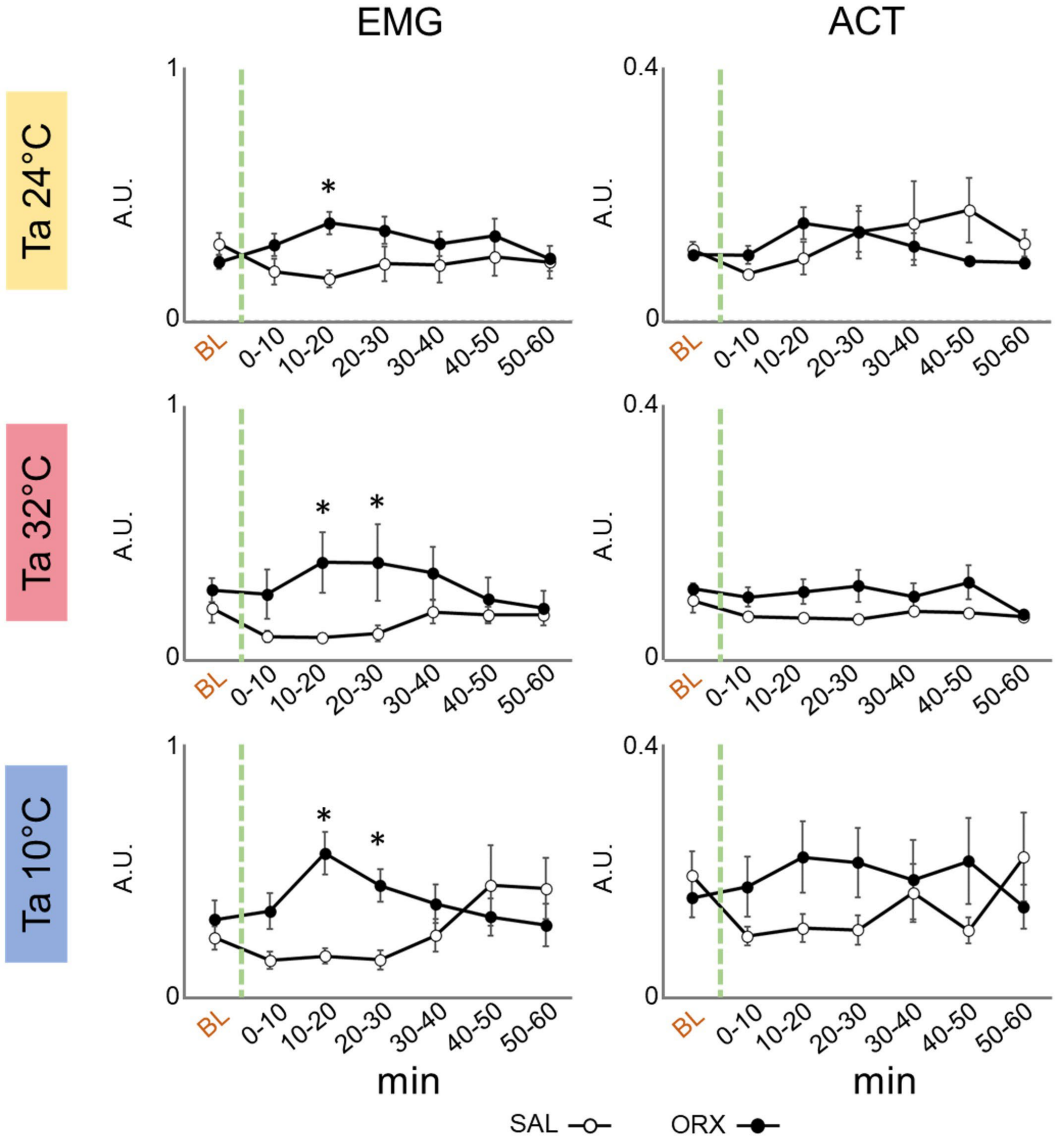
Graphs depicting the mean value ± S.E.M. of nuchal electromyographic activity (EMG) and movement in the cage (ACT), for the 60 min after the injection (green dashed line) of either saline (SAL, empty dots), or orexin (ORX, filled dots) in the Raphe Pallidus, at three different ambient temperatures (Ta). BL = baseline, indicates the mean value ± S.E.M. of the 30 min prior to the injection. The 60 min following the injection are showed in 10-min time bins. * = *p* < 0.05, green dashed line = injection time, A.U. = arbitrary units.

We assessed motor activity by means of electromyographic (EMG) electrodes to detect nuchal muscles activity and a passive infrared detector to detect the movement in the cage. As shown in Figure 4, a significant increase in EMG activity was observed 10–20 min after orexin compared to saline administration that consistently peaked across the three Ta. On the contrary, we did not observe any change in the activity measured by means of the infrared detector, showing that orexin injection in the RPa did not significantly affect the amount of movement in the cage.

### Effects on feeding behavior

**Figure 5:**
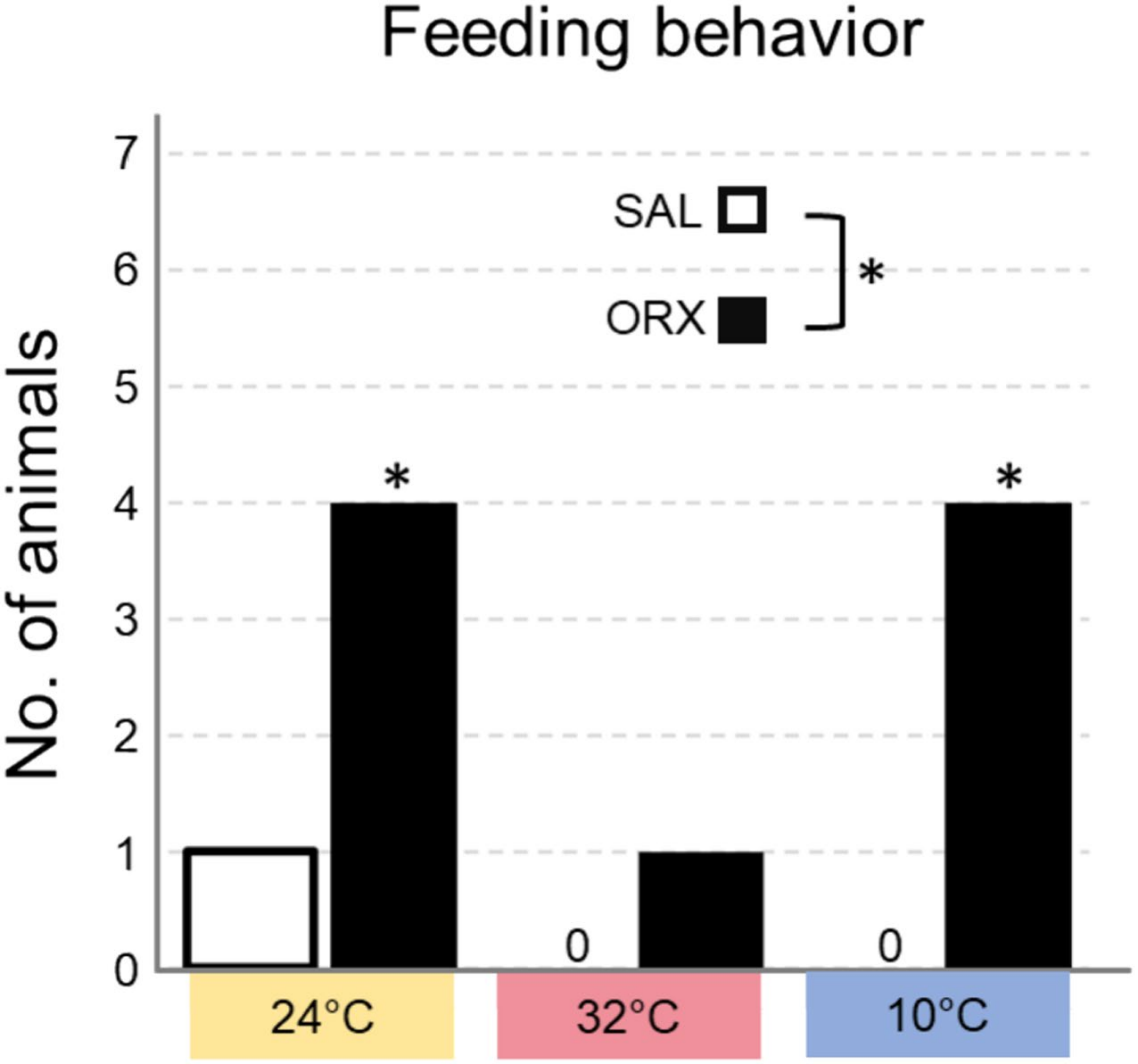
Number of animals that consumed food in the 60 min following the injection of either saline (SAL) or orexin (ORX), in the Raphe Pallidus, at three different ambient temperatures. * = *p* < 0.05, SAL vs. ORX.

We also assessed whether the animals displayed feeding behavior in the 60 min following the injection, and categorized the results in yes/no dichotomous variables (Figure 5). The experiment was carried during lights on, corresponding to the rats’ rest phase, a time when they rarely consume food spontaneously. Overall, orexin injection enhanced feeding behavior at the different Ta compared to saline and feeding occurrence was different at the three Ta, but no significant interaction between the two treatments was observed. *Post hoc* analysis of feeding occurrence at the three Ta showed that the difference observed between ORX and SAL was significant at both Ta 24°C and 10°C, but not at Ta 32°C.

## Discussion

The results of this study corroborate existing literature on how orexin-A in the RPa modulates cardiovascular responses and thermogenesis, enhancing an already present thermogenic drive under anesthesia (Tupone et al., 2011). Additionally, our findings provide new insights into the effects of orexin injection into the RPa, which induced significant changes in wake–sleep states, autonomic functions, motor activity, and feeding behavior, with varying effects depending on the Ta.

The most intriguing aspect of our study is the wake-promoting effect of orexin administration in the RPa of freely behaving rats, with the strength of this effect varying depending on the Ta, since we observed that the increase in wakefulness was more pronounced at Ta 24°C and Ta 10°C compared to Ta 32°C.

Although the role of orexin in promoting wakefulness is well established, this finding was rather unexpected: it is known that hypothalamic orexinergic neurons project an intricate network of efferences to cortical and subcortical areas, notably wake-promoting regions such as the locus coeruleus, dorsal raphe, ventral tegmental area, and tuberomammillary nucleus. These orexinergic projections also directly and indirectly activate cortical neurons, via effects in the basal forebrain (Alexandre et al., 2013). However, until now, the RPa has not been described as part of this wake-promoting circuitry. Even though some sparse staining was observed in RPa when injecting a retrograde tracer in the locus coeruleus (Morgane and Jacobs, 1979) RPa neuronal populations primarily project downstream and therefore are not apparently directly capable of modifying cortical activation states. This could mean that the peripheral autonomic activation induced by RPa could influence the activity of hypothalamic integrative centres responsible for determining the vigilance state as a neurovegetative reflex, meaning that the activation of the sympathetic nervous system could inherently result in cortical activation as a consequence.

However, the autonomic activation observed following orexin administration in the RPa is evident only at Tas of 24°C (increase in ABP) and 10°C (increase in Thy and in HR), with no discernible effects at 32°C, despite the consistent increase in wakefulness observed across the three Tas. This discrepancy may stem from the limitations of the variables we monitored, potentially rendering sympathetic activation at Ta 32°C undetectable despite its presence, or it may indicate a nuanced scenario. It is possible that orexin administration in the RPa triggers both central and peripheral responses. On the one hand, although there is scarce evidence about excitatory projections from RPa to arousal-promoting centres, we cannot exclude that RPa could be acting centrally through its efferences, causing an autonomic activation. On the other hand, the observed increase in sympathetic activity at Ta 24°C and Ta 10°C could initiate a feedback loop, promoting wakefulness. Hence, these dual mechanisms possibly synergize, acting as co-factors in promoting vigilance. It is also worth considering that although RPa is known to be involved in the central control of pain (Heinricher et al., 2004; Mason, 2001), given that injection of orexin-A in the RPa has shown to have antinociceptive properties (Azhdari-Zarmehri et al., 2014), the increase in wakefulness is most likely not related to an increase in pain perception. Further experiments are needed to elucidate these questions.

Regarding thermoregulation, experiments in anesthetized rats proved that orexin is able to potentiate the ongoing activity in the RPa premotoneurons resulting in an amplified thermogenic response (Tupone et al., 2011). Based on these findings, orexin alone would have little effect in the presence of a reduced or absent excitatory tone to RPa neurons, while it would be more effective when the organism is already engaged in thermogenic activity. Our data obtained from freely moving, non-anesthetized rats confirm these results. The observed increase in central temperature was relevant at Ta 10°C, and absent at Ta 24°C and Ta 32°C, respectively, confirming that, as shown by Tupone et al. (2011), orexin alone is not able to trigger an effective thermogenic response from RPa neurons in the absence of other excitatory neurotransmitters or in the presence of a high inhibitory tone.

We also did not observe any effect on cutaneous vasomotion at any Ta. Although this could be interpreted as orexin works on only on RPa premotor neurons controlling BAT or shivering thermogenesis, and so might not have a role/influence on the modulation of cutaneous vasomotion, we believe that, given the amplifying role that orexin has on ongoing thermogenic drive (Tupone et al., 2011), we should expect a similar modulatory effect on RPa premotoneurons controlling vasomotion. However, at Ta 10°C, since vasoconstriction is already at its maximum at low Ta, any further potentiation of vasoconstrictor tone mediated by orexin would not be noticeable, making it likely for the tail to remain vasoconstricted.

As expected, we did not observe any increase in vasoconstrictor tone neither at Ta 24°C, nor in vasodilated animals at 32°C. This is likely due to the inability of orexin to exert a potentiating effect in the presence of reduced or absent ongoing activity from RPa premotoneurons controlling vasomotion.

Cardiovascular function is influenced by orexin delivery in the RPa with regard to ABP regulation. At Ta 24°C, we observed a significant increase in mean ABP, which was not replicated at Ta 32°C or Ta 10°C. The lack of effects at Ta 32°C may be attributed to the absence of an excitatory tone or to an increased inhibitory tone on RPa neurons caused by the exposure to a high Ta. Exposure to Ta 10°C, on the other hand, causes *per se* an elevation in baseline ABP (mean ± S.E.M.: SAL 117 ± 2 mmHg; ORX 116 ± 1 mmHg), compared to Ta 24°C (mean ± S.E.M.: SAL 103 ± 2 mmHg; ORX 101 ± 1 mmHg) and 32°C (mean ± S.E.M.: SAL 92 ± 2 mmHg; ORX 93 ± 2 mmHg), it is therefore possible that high ABP levels limit further increases of such parameter. The observed increase at Ta 24°C could therefore be the consequence of the strong behavioral activation observed in the animal after orexin injection.

Overall, the effects on HR of orexin delivery were practically absent. This finding is interesting because the activation of RPa neurons is known to exert a significant enhancing effect on HR in anesthetized rats (Cerri and Morrison, 2005). It is possible that the population of neurons targeted by orexin in the RPa is not the same as those activated following various pharmacological stimulations of these neurons. For example, the administration of the GABA_A_ receptor antagonist bicuculline into the RPa produces a massive increase in HR (Cao and Morrison, 2003), as does the administration of orexin (Tupone et al., 2011). The activation of thermogenesis evoked by higher nervous centers, such as it was observed following the administration of the GABA_A_ receptor antagonist bicuculline into the DMH (Samuels et al., 2004; Zaretskaia et al., 2008), or NMDA in LH (Tupone et al., 2011) produces significant increases in HR mediated by the RPa. Although specific to receptor type, the pharmacological stimulations described can act on different neuronal populations, as GABA_A_ and NMDA receptors are widespread.

The inefficacy of orexin in causing an increase in HR suggests that orexin itself may act on a specific subset of neurons in the RPa. In particular, the population of serotoninergic neurons in the RPa could be an excellent candidate target for orexin action. These serotoninergic neurons are not tonically active, and no effects were reported following the administration of a 5-HT1A receptor antagonist into the RPa. These neurons have also been identified as the only ones in the area possessing receptors for a strong pyrogen such as prostaglandin E2 (Nakamura et al., 2000). On these grounds, it could be hypothesized that the effect evoked by orexin in the RPa differs from what is observed following activation of different neuronal subgroups in the area. Immunohistochemical analysis could verify if serotoninergic neurons in the area are the only ones expressing orexin receptors and being activated following its administration.

The impact on motor activity is worth noting. While the extended wakefulness might imply heightened motor activity, intriguingly, we did not observe significant differences in movement within the cage between groups, as recorded by the passive infrared detector. Nevertheless, we did observe a surge in nuchal electromyographic activity following orexin administration at the three Ta. Among the many possibilities that could explain this phenomenon, we speculate a heightened sympathetic tone through efferences from the RPa and directed at nuchal muscles, or the activation of specific behaviors such as rearing or food consumption, that involve the use of nuchal muscles.

The observed effects on feeding behavior demonstrate that orexin administration does indeed promote feeding regardless of Ta. However, the *post-hoc* analysis show that the increase in feeding behavior was a significant at Ta 10°C and Ta 24°C, but not at Ta 32°C. This could be ascribed to a mechanism similar to what was discussed for the observed increase in time spent in wakefulness: orexin might be able to amplify an already existing arousal-promoting sympathetic tone, but it produces more nuanced responses when sympathetic activity is very low, such as at Ta 32°C. Therefore, the increase of food consumption could be considered as an additional output to measure the effects of orexin in promoting arousal. An alternative interpretation may be rooted in the activity of gastrointestinal organs downstream of RPa neurons. Anatomically, neurons within this region have been shown to be multi-synaptically connected with the liver (Kalsbeek et al., 2004), and the pancreas (Streefland et al., 1998). Although there is no indication of the functional role of these connections, their role in promoting feeding cannot be ruled out. How the increase in feeding behavior corresponds to an actual increase in food intake remains unexplored, and could be investigated in future experiments aimed at understanding the role of orexin signalling in RPa in food consumption.

In conclusion, this work furthers our knowledge on the behavioral and autonomic effects of orexin signalling in the RPa. The findings demonstrate that these effects vary with Ta, reinforcing the finding that orexin is unable to trigger an autonomic response in the absence of an already existing thermogenic drive previously observed in anesthetized animals (Tupone et al., 2011). Furthermore, it introduces the RPa as a novel player in the central neural network that promotes wakefulness. Further research, particularly involving immunohistochemical analyses, is necessary to elucidate which neural populations are targeted by orexin in the RPa and to clarify the mechanisms underlying its complex effects on behavioral and autonomic functions.

## Data Availability

The raw data supporting the conclusions of this article will be made available by the authors, without undue reservation.
